# Considerations for first field trials of low-threshold gene drive for malaria vector control

**DOI:** 10.1186/s12936-024-04952-9

**Published:** 2024-05-22

**Authors:** John B. Connolly, Austin Burt, George Christophides, Abdoulaye Diabate, Tibebu Habtewold, Penelope A. Hancock, Anthony A. James, Jonathan K. Kayondo, Dickson Wilson Lwetoijera, Alphaxard Manjurano, Andrew R. McKemey, Michael R. Santos, Nikolai Windbichler, Filippo Randazzo

**Affiliations:** 1 Department of Life Sciences, Silwood Park, Imperial College London, London, UK; 2 Department of Life Sciences, South Kensington Campus, Imperial College London, London, UK; 3 Institut de Recherche en Sciences de la Santé/Centre Muraz, Bobo-Dioulasso, Burkina Faso; 4 Environmental Health and Ecological Science Department, Ifakara Health Institute, Ifakara, Tanzania; 5 MRC Centre for Global Infectious Disease Analysis, St. Mary’s Campus, Imperial College London, London, UK; 6 Departments of Microbiology & Molecular Genetics and Molecular Biology & Biochemistry, University of California, Irvine, USA; 7 Entomology Department, Uganda Virus Research Institute (UVRI), Entebbe, Uganda; 8 Malaria Research Unit and Laboratory Sciences, Mwanza Medical Research Centre, National Institute for Medical Research, Mwanza, Tanzania; 9 Foundation for the National Institutes of Health, North Bethesda, MD USA; 10 Leverage Science, LLC, Berkeley, CA USA

**Keywords:** Adaptive trial design, Africa, *Anopheles*, Cluster randomized controlled trial (cRCT), Genetic efficacy, Entomological efficacy, Epidemiological efficacy, Mosquito, Population modification, Population suppression, Spillover

## Abstract

Sustainable reductions in African malaria transmission require innovative tools for mosquito control. One proposal involves the use of low-threshold gene drive in *Anopheles* vector species, where a ‘causal pathway’ would be initiated by (i) the release of a gene drive system in target mosquito vector species, leading to (ii) its transmission to subsequent generations, (iii) its increase in frequency and spread in target mosquito populations, (iv) its simultaneous propagation of a linked genetic trait aimed at reducing vectorial capacity for *Plasmodium*, and (v) reduced vectorial capacity for parasites in target mosquito populations as the gene drive system reaches fixation in target mosquito populations, causing (vi) decreased malaria incidence and prevalence. Here the scope, objectives, trial design elements, and approaches to monitoring for initial field releases of such gene dive systems are considered, informed by the successful implementation of field trials of biological control agents, as well as other vector control tools, including insecticides, *Wolbachia,* larvicides, and attractive-toxic sugar bait systems. Specific research questions to be addressed in initial gene drive field trials are identified, and adaptive trial design is explored as a potentially constructive and flexible approach to facilitate testing of the causal pathway. A fundamental question for decision-makers for the first field trials will be whether there should be a selective focus on earlier points of the pathway, such as genetic efficacy via measurement of the increase in frequency and spread of the gene drive system in target populations, or on wider interrogation of the entire pathway including entomological and epidemiological efficacy. How and when epidemiological efficacy will eventually be assessed will be an essential consideration before decisions on any field trial protocols are finalized and implemented, regardless of whether initial field trials focus exclusively on the measurement of genetic efficacy, or on broader aspects of the causal pathway. Statistical and modelling tools are currently under active development and will inform such decisions on initial trial design, locations, and endpoints. Collectively, the considerations here advance the realization of developer ambitions for the first field trials of low-threshold gene drive for malaria vector control within the next 5 years.

## Background

Insecticide-treated bed nets (ITNs) and indoor residual insecticide spraying (IRS) are currently the main tools for malaria vector control, which mainly act on vectorial capacity by reducing the density and longevity of adult female mosquitoes [1, 2]. While new insecticides, spatial repellents, housing modifications, and mosquito traps are in the research and development pipeline, both the WHO and African Union have recognized that these will be insufficient to meet the challenges of reducing the malaria burden and new transformational tools will be required along with additional deployment of existing interventions [3, 4]. Among such tools, genetic approaches, including gene drive systems that target the mosquitoes that transmit malaria parasites are currently being developed and evaluated for potential use in the field.

Gene drive systems promote the biased inheritance of specific genes from one generation to the next [5–8]. While several disparate mechanisms exist, the biased inheritance of the most well-studied type is achieved via a process known as ‘homing’ that exploits CRISPR biology [8]. Here the gene drive system is (i) inserted at a specific genomic target locus on one of a pair of homologous chromosomes and encodes both (ii) the Cas endonuclease under control of a promoter with germline activity and (iii) a guide RNA that targets the specific genomic locus into which the gene drive system is inserted and homes. In germline cells, the Cas/gRNA complex causes a double-strand break in the genomic target locus on the homologous chromosome where the gene drive system is absent [7–9]. Homology-directed repair uses the chromosome encoding the gene drive system as a template to repair that double-stranded break and in doing so, copies the gene drive system from the intact chromosome onto the cleaved homologous chromosome.

The net result of this homing reaction is that both copies of homologous chromosomes in the germline contain the gene drive system, so that most gametes, and consequently most progeny, will possess a copy of gene drive system at the genomic target site. Importantly, homing of a gene drive system also can be associated with the introduction into mosquito target populations of a genetic trait that reduces vectorial capacity, by either reducing the density of *Anopheles* females in the case of “population suppression”, or impeding their ability to transmit *Plasmodium* parasites, in “population modification”, also called “population replacement”. Thus, *genetic efficacy* is used to refer to the efficiency in the field with which the gene drive system can increase in frequency, or proportion, while simultaneously introducing genetic traits that can reduce vectorial capacity, in target mosquito populations at release locations, as well as spread into target mosquito populations at distal locations [10].

**Fig. 1:**
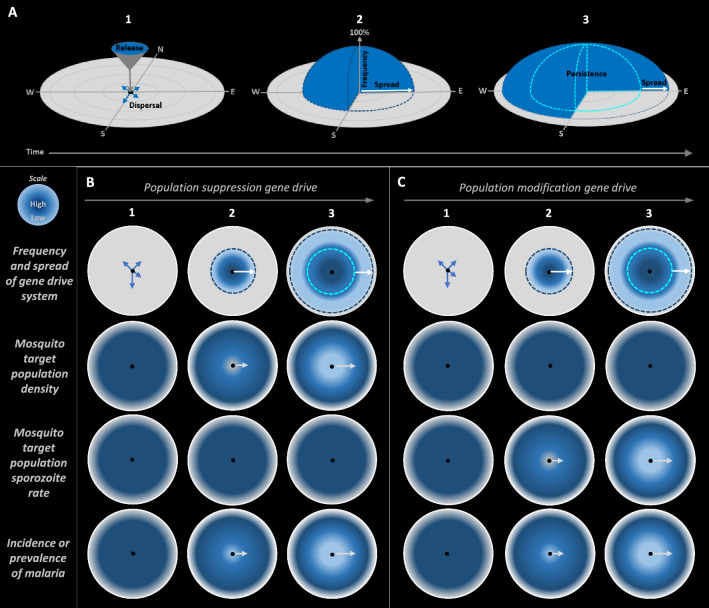
Conceptual diagram illustrating temporal and spatial dynamics of gene drive systems. **A1** Gene drive mosquitoes are released at specific release locations (black dots) and individual mosquitoes *disperse* to different habitats [12, 110, 167]. **A2** Over time and multiple generations, the gene drive progressively increases in *frequency*, (measured as a percent, or proportion, of gene drive mosquitoes from the total mosquito population) and, aided by the dispersal of individual mosquitoes to different habitats, *spreads* to other individuals through interbreeding in target populations (white arrow) [12]. **A3** The gene drive *persists* in this area (dome within light-blue dotted borders) and continues to *spread* further through target populations (white arrow). **B1** In the case of population suppression gene drive, the gene drive system is released in mosquitoes which disperse from release locations. **B2** The gene drive system increases in frequency at release locations, observed as an increase in frequency of mosquitoes heterozygous for the gene drive system. The gene drive also spreads to distal target populations, increasing in frequency as it does so. Increasing numbers of mosquitoes that are heterozygous for the gene drive leads to increasing chances of heterozygous males mating with heterozygous females, thus increasing the number of female progeny that are homozygous for the gene drive system, and thus sterile. **B3** This leads to a progressive reduction in the density of the target mosquito populations, propagating out over time from release locations, with the spectrum of navy to sky blue indicating high to low population densities in the illustration. These progressive reductions in vector numbers would correlate with progressive reductions in the incidence of malaria, with the spectrum of navy to sky blue indicating high to low malaria incidence in the illustration, and the progressive nature of these effects indicated by grey arrows. No direct change in the prevalence and intensity of infectious *Plasmodium* sporozoites in mosquitoes would be anticipated initially, although over time with anticipated reduced prevalence of malaria in humans, sporozoite rates in mosquitoes would be reduced. The frequency of the gene drive system in the mosquito population should be unaffected by its suppression, unless the population was to be eliminated completely. **C1** For population modification gene drive, the gene drive system is released in mosquitoes which disperse from release locations. **C2** The gene drive system increases in frequency. **C3** This would be associated with progressive decreases in sporozoite rates in target populations of mosquito, leading to progressive reductions in the incidence of malaria, without any anticipated changes in the densities of mosquito target populations

While disparate gene drive systems can be designed to have a range of levels of increase in frequency, spread and persistence in target populations, it is the self-sustaining, non-localizing characteristics inherent in “low threshold” gene drive systems that would be expected to require minimal numbers of mosquitoes to be released for achieving efficacy in the field, making them particularly attractive for malaria control. Low-threshold gene drive systems are designed to progressively increase in frequency and spread indefinitely, in every generation post-release, and eventually persist at stable levels (Fig. 1) [6, 10, 11].

As low-threshold gene drive systems reach fixation in target populations, they are intended to meet the prerequisites for a novel vector control tool by: (i) reducing the vectorial capacity of target mosquito populations for *Plasmodium* transmission, referred to here as *entomological efficacy*, and (ii) reducing the incidence and prevalence of malaria in humans, referred to here as *epidemiological efficacy*, while being effective over wide geographic areas, acting at low cost, and not requiring human behavioural change. Low-threshold gene drive systems could, therefore, be deployed to help reduce transmission rates in high transmission areas, particularly in areas where other more human-intensive delivery methods are challenging, or in low transmission areas to help accelerate malaria elimination, or to help prevent reintroduction and transmission in areas that have been declared malaria-free [4].

Over 2022 and 2023, the GeneConvene Global Collaborative of the Foundation for the National Institutes of Health (FNIH) assembled a group of developers of low-threshold gene drive systems for malaria vector control that are considered to be at the closest stages of readiness for proposals of field-testing. The group considered some of the key challenges, specific efficacy and risk research questions, knowledge gaps, and potential solutions that would apply to initial field release trials, building on the ‘Guidance framework for testing genetically modified mosquitoes’ (GMMs) of the WHO [12]. The term “trial” was used in its broadest context here, analogous to the range of designs typically found in pharmaceutical development programmes, from Phase 0 trials assessing pharmacodynamic and pharmacokinetic endpoints or Phase I dose-finding trials, both of which do not involve controls, to Phase II trials assessing the intervention compared to a placebo control group, through to Phase III trials often involving comparison with the standard of care [13]. Informed by a broad range of consultations with other experts in entomology, epidemiology, and vector control field trials, the outcomes of those deliberations are presented here.

Box 1. Governance, oversight, and guidance Decisions on the first field trials of low-threshold gene drive for malaria vector control in Africa will require the submission by developers to national regulatory authorities in African countries of field trial applications and protocols that have been previously developed in dialogue with other stakeholders such as affected communities and funders. Governance and oversight will primarily occur via national and institutional ethical, biosafety, and environmental oversight mechanisms that have been developed for all applications of genetically modified organisms, and which is addressed by national legislation that has typically been derived from international instruments such as the Cartagena Protocol on Biosafety for the Convention on Biological Diversity [19, 20]. This oversight is grounded in rigorous testing of efficacy and safety parameters in the laboratory that inform environmental risk assessment (ERA), and other environmental and social impact assessments, before any releases in the field can occur [19–21].

Voluntary guidance frameworks on the evaluation of genetically modified mosquitoes (GMMs) have also been published both by the WHO [12], and jointly by the African Union Development Agency-New Partnership for Africa's Development (AUDA-NEPAD) and the West African Health Organization (WAHO) via their West African Integrated Vector Management (WAIVM) programme [22]. A continuum of research is proposed in both guidance frameworks, based on a phased testing approach that starts with safety and efficacy testing of the GMM in indoor laboratory facilities and insectary population cages as Phase 1; leading to small-scale Phase 2 releases measuring entomological efficacy and with some degree of isolation that has been informed by risk assessment; to open field releases in Phase 3 measuring epidemiological efficacy; culminating in post-implementation surveillance in Phase 4. However, in the case of low-threshold gene drive systems, it is also recognized that the potential for indefinite spread and persistence makes a clear delineation between Phase 2 and later Phases impractical, so that field testing could be perhaps better conceived of as a spectrum of expanding releases (Fig. 3) [12]. Both WHO and WAIVM guidance frameworks propose that the decision for environmental release of any GMMs must be preceded by careful evaluation of their potential safety and efficacy in the field, considering both benefits and risks [12, 22, 23]. This is consistent with governance of biological control agents that also possess the potential for indefinite spread and persistence. Before any such field releases, the International Plant Protection Convention, overseen by the Food and Agricultural Organization of the United Nations, advocates rigorous science-based ERA based on International Standards for Phytosanitary Measures, and numerous jurisdictions have established national regulatory systems based on this approach [24–30].

For trials involving human participants, including gene drive field studies that would assess epidemiological endpoints, field trial design and implementation also will be shaped by adherence to fundamental standards, such ethics approval underpinned by the Declaration of Helsinki of the World Medical Association [31], guidelines from the International Council for Harmonization (ICH) on Good Clinical Practice (GCP) [32], and requirements for an independent Data and Safety Monitoring Board (DSMB) [33, 34]. Additionally, the International Committee of Medical Journal Editors has stipulated that submission for publication of ‘*any research study that prospectively assigns human participants or groups of humans to one or more health-related interventions to evaluate the effects of health outcomes*’ should first be reported on a public clinical trials register before any participant is enrolled [34]. Moreover, where personally identifiable information would be collected as part of a field trial, protections of the rights to privacy and anonymity of human participants would need to be addressed.

Subsequent to initial field trials, for low-threshold gene drive to be used as a vector control intervention by agencies of the United Nations, WHO, WHO member states, and many philanthropic bodies, it must first be “pre-qualified” by the WHO [17]. This requires a WHO recommendation based on assessments both of the safety, quality, and entomological efficacy of the intervention by the WHO Prequalification Team for Vector Control Products, and of the epidemiological efficacy by VCAG [17]. VCAG has previously published guidance on the design of vector control field trials to facilitate this process [15]. Initial and subsequent field trials of low-threshold gene drive would need to be designed and implemented with these WHO requirements for assessments in mind.

## Trial objectives

**Fig. 2:**
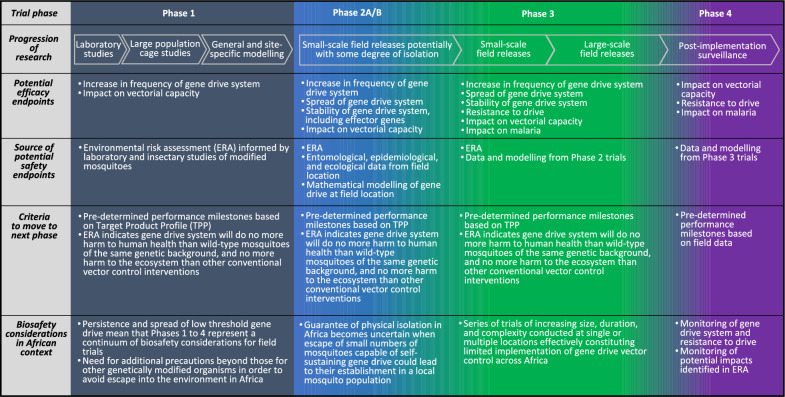
Phased testing pathway for low-threshold gene drive system from laboratory studies to post-implementation surveillance, as recommended by the WHO [12]. In the case of low-threshold gene drive systems, it is recognized that the potential for indefinite spread and persistence makes a clear delineation between Phase 2 and later Phases impractical, so that field testing could be perhaps better thought of as a spectrum of expanding releases [12]. Therefore, safety testing in Phase 1 laboratory, insectary, and modelling studies along with thorough risk assessment prior to Phase 2 will be particularly important for low-threshold gene drive applications, as has been the case for releases of *Wolbachia* and biological control agents. As highlighted in the main body of this article and Fig. 3, Phase 2 could be further subdivded into different trial phases whereby Phase 2A would measure genetic efficacy and Phase 2B would measure entomological efficacy

**Fig. 3:**
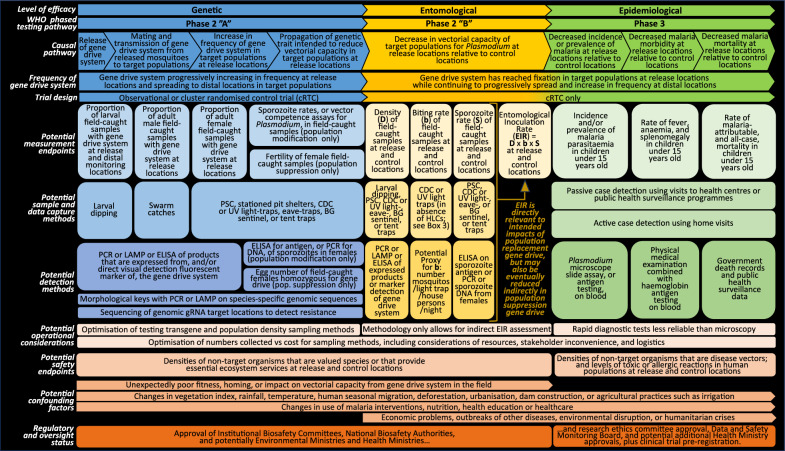
Potential design features for initial field trials of low-threshold gene drive systems. There are three potential levels of efficacy, which could be assessed separately or in different combinations in initial field trials. Where genetic efficacy (WHO Phase 2 “A”; [12]) would be the sole efficacy objective of initial field trials, this could potentially be assessed in observational studies without control locations, given the pre-release absence of the gene drive system from wild target mosquito populations, provided potentially confounding factors such as rainfall were recorded. Where the objectives of an initial field trial were also to assess entomological (WHO Phase 2 “B”) and epidemiological efficacy (WHO Phase 3), cRCTs would provide the most robust estimates of entomological and epidemiological efficacy with least opportunity for introduction of bias from confounding factors. Genetic efficacy of a low threshold gene drive system involves its increase in frequency at, and spread from, the release location in target populations. This could be measured using a variety of endpoints and field methodologies as outlined. Entomological efficacy could be established by measuring the impact of the low threshold gene drive on vectorial capacity using highlighted endpoints and methodology. Epidemiological efficacy could be measured as impact on rate of infection or malaria incidence and prevalence, as indicated. The range of endpoints chosen for initial field trials, whether addressing genetic, entomological, or epidemiological efficacy, will depend on power calculations for specific field methods underpinning them, as well as operational and cost considerations. In addition to efficacy assessments, considerations of the design of initial field trials may consider operational issues, the potential to assess potential safety endpoints, in addition to capturing data and information before and during trials on potential confounding factors that could impact efficacy assessments. Furthermore, considerations of the scope of efficacy measurements will also need to consider the higher levels of oversight and governance that accompany epidemiological efficacy assessment as clinical trials

For a low threshold gene drive system to be successful as a malaria vector control tool in the field, a ‘causal pathway’ would be initiated by (i) the release of a gene drive system in target mosquito vector species, leading to (ii) its transmission to subsequent generations, (iii) its increase in frequency and spread in target mosquito populations, (iv) its simultaneous propagation of a linked genetic trait aimed at reducing vectorial capacity for *Plasmodium*, and (v) reduced vectorial capacity for *Plasmodium* in target mosquito populations as it reaches fixation in target mosquito populations, resulting in (v) decreased malaria incidence and prevalence (Fig. 3). Therefore, assessments of the efficacy of some or all of this causal pathway will be key objectives for initial field trials of low-threshold gene drive. Choices on objectives of those initial field trials will fundamentally inform their design so that explicit decisions will be required a priori on whether the focus and design for these initial field release trials should rest exclusively on assessments of genetic, entomological, or epidemiological efficacy, or on combinations thereof [12, 14–18]. In the WHO guidance framework for testing GMMs, it is envisaged that both genetic efficacy and entomological efficacy would be tested in Phase 2, following the successful completion of Phase 1 laboratory, insectary, and modelling studies [12]. To maintain consistency with WHO terminology, assessment of genetic efficacy as proposed here could occur in Phase 2 “A” of initial field trials, entomological efficacy at Phase 2 “B”, and epidemiological efficacy at Phase 3 (see Box 1 and Fig. 2).

The design of initial field trials also will be determined by safety objectives [12, 19, 20, 22]. Before regulatory applications for initial field trials can be made, both efficacy and safety of low-threshold gene drive must first be assessed in the laboratory [12] (Box 1). Safety endpoints will be informed by pre-release environmental risk assessment (ERA) on a case-by-case basis depending on the gene drive systems being used and on the choice of study locations [12, 22] (Box 2). For example, population suppression gene drive research questions could include whether there were any adverse impacts of the gene drive system on non-target organisms, or on competitor or predator species, that could impinge on health or environmental protection goals (Table 1). By contrast, population modification questions might be centered around the potential for *Plasmodium* resistance to the activity of effector gene or increased vector competence for a non-target pathogen. Moreover, even after the primary objectives of the first field trials have been met, longer-term monitoring of efficacy and safety at release and control locations may continue based on outputs from the ERA [12]. Related to this, the impacts of the releases on stakeholder perceptions will represent fundamental considerations on the acceptability of gene drive systems in affected communities. Therefore, pre-, during-, and post-release measurement and assessment of such perceptions should also form an integral aspect of the design of initial field trials of low-threshold gene drive for malaria vector control.

**Table 1:** Defining specific research questions for programmes assessing low-threshold gene drive systems in initial field release trials

Analysis element of causal pathway	Broad measurement endpoints	Specific research questions	
		Population suppression	Population modification
Genetic efficacy	A. Extent of increase in frequency of the gene drive system in target mosquito populations	1. Do the released mosquitoes carrying the gene drive system mate with the target population and is the gene drive system successfully introduced into wild populations? 2. Does the gene drive system increase in frequency as expected over successive generations? 3. Is there any evidence of the emergence of alleles that are resistant to gene drive?	
	B. Rate of spread from release locations of the gene drive system in target mosquito populations	4. What is the rate of spread of the gene drive system in the target population?	
	C. Extent that the gene drive system alters vectorial capacity in mosquito target field populations	5. Is the main phenotype, that females homozygous for the gene drive system are sterile, observable in the field and does this increase in prevalence as the gene drive system increases in frequency?	5. Does the main phenotype, that females carrying the gene drive system impede *Plasmodium falciparum* development, increase in prevalence as the gene drive system increases in frequency?
Entomological efficacy	D. Extent that the introduction of the gene drive system coincides with any changes in target mosquito populations or their parasites	6. Does the increasing female sterility caused by the gene drive system lead to reduced densities of the target population? 7. Is there a change in the EIR of the target populations before and after field releases or at release versus control locations? 8. Is there any evidence of the emergence of alleles that are resistant to population suppression? 9. Is the gene drive system stable over time?	6. Does the diverse genetic background of field-collected mosquitoes affect the expected *Plasmodium falciparum* transmission-blocking phenotype? 7. Is there a change in the EIR of the target populations before and after field releases or at release versus control locations? 8. Is there any evidence of the emergence of resistance to the effector gene in *Plasmodium falciparum* or in mosquitoes? 9. Is the gene drive system stable over time, such that the gene drive remains linked to the functional effector gene?
Epidemiological efficacy	E. Extent that the introduction of the gene drive system coincides with any changes in disease incidence or prevalence	10. Is there a change in the incidence or prevalence of malaria before and after field releases or at release versus control locations?	10. Is there a change in the prevalence or incidence of malaria before and after field releases or at release versus control locations?
Risk, Entomological efficacy, Epidemiological efficacy,	F. Extent that the risk profile from the releases of the gene drive system is consistent with the outputs of the pre-release ERA	11. Does the gene drive system spread to other sibling species that are sympatric to the released transgenic species and causing adverse impacts? 12. Is any adverse impact of the gene drive system detected on non-target organisms that impinges health or ecosystem services? 13. Is any adverse impact of population suppression detected on competitor or predator species that impinges health or ecosystem services? 14. Is there any evidence of gene flow into, and population suppression of, non-reproductively compatible species of insect and that causes adverse impacts?	11. Does the gene drive system spread to other sibling species that are sympatric to the released transgenic species and causing adverse impacts? 12. Is any adverse impact of the gene drive system detected on non-target organisms that impinges health or ecosystem services? 13. Are there any changes correlated with reduction in *Plasmodium falciparum* in other species of malaria pathogens such as *Plasmodium vivax*, *Plasmodium ovale*, or *Plasmodium malariae* that adversely impacted health or ecosystem services? 14. Is there any evidence of gene flow into non-reproductively compatible species of insect and that causes adverse impacts?
Stakeholders, Epidemiological efficacy	G. Impacts of the field release trials on local or wider stakeholder perspectives to gene drive	15. Do the releases and their impacts change the perspectives of the local or wider communities to the specific intervention and wider technology?	

Research questions presented here should be considered as illustrative examples rather than an exhaustive list. Some research questions presented here may be appropriate for some field trial designs and not others, depending on trial objectives

Box 2. Choice of trial location A key element in the implementation of the first field trials for low-threshold gene drive will be the judicious selection of field trial locations [35]. There are at least five broad criteria that will collectively contribute to such decisions:

(*1) Operational viability*

This would include the credibility of the regulatory structure at the location to allow decision-making on environmental releases of GMMs containing gene drive systems, availability of adequate current and historical public health surveillance data, availability of adequate control sites near to proposed release sites, lack of adverse climatic or environmental conditions, the safety profile of the trial location for project staff, the presence of local expertise in vector biology and field epidemiology, stakeholder perceptions on field testing of GMMs, and outcomes of previous experiences with applications involving genetic modification, the accessibility of the proposed location, the ability to deploy the resources required for field studies, including the production and transportation of mosquitoes to be released, collection of field samples and their delivery for laboratory processing, and availability of reliable molecular assays.

*(2) Species composition*

Consideration should be given to mosquito species composition in the trial area. Measurement of both entomological and epidemiological efficacy is likely to be more complicated where the gene drive system is released in a single species but where there are multiple species of *Anopheles* malaria vectors at the release location. Therefore, any change in the EIR of target populations from release of the gene drive system could be partially confounded by continuing pathogen transmission by other sibling species, or by other malaria vector species, such as *Anopheles funestus* [36–38]. Modelling may help to predict the full impact if these other vector species are also targeted as there may be non-linear relationships between species abundance and malaria prevalence as transmission drops to low levels. It is also plausible that the gene drive system could introgress into other sibling species of the *An. gambiae* species complex. For instance, hybrids of sibling species have been detected, albeit rarely, in the field [39–45], and some genomic studies have indicated gene flow among sibling species [46–49]. Direct measurements in field trials may help to determine the precise probability that vertical transfer of the gene drive system will occur among sibling species in the field.

*(3) Statistical power*

Initial field release trials by their very nature are likely to be relatively small so that some locations will provide the opportunity for greater statistical power than others. For example, choice of a study location with low levels for malaria transmission may provide insufficient statistical power because cases of infection could be too rare to detect epidemiological impacts from vector control, while in locations where malaria transmission is high, the epidemiological impact of the intervention could be overshadowed. The scale, size, and duration of the proposed field release study as well as levels and variability of seasonality will affect statistical power and therefore choice of trial location. Considerations of statistical power also may differ according to whether genetic, entomological, or epidemiological endpoints are chosen. For example, proposed study locations with multiple vector species may offer more statistical power by allowing entomological endpoint comparisons of target vector species with non-target species that could act as controls.

*(4) Degree of isolation*

WHO guidance suggests that the first field release studies of gene drive systems could aim for some potential form of geographical isolation of the target mosquito population, informed by ERA, to minimize the potential for outward migration of the gene drive system, as well as limiting inward migration that could potentially confound interpretations of efficacy [12]. Initial field release studies of GMMs have previously been proposed on geographic islands, including off the coast of continental Africa for low-threshold gene drive [50–54]. While genetic analysis of mosquito populations on islands such as São Tomé [54] show significant differentiation from those on the mainland that might support such proposals, it remains possible that human transport links, via air travel and shipping, or high-altitude mosquito migrations could facilitate spread of gene drive systems to varying degrees from different island settings to mainland Africa [55]. Indeed, the WHO guidance acknowledges that preventing outward or inward migration cannot be fully guaranteed in an island context. Moreover, limiting inward migration in an island setting means that the intervention may perform differently in island and continental settings [50]. Plausible intermediate approaches to coastal islands could involve island settings within continental Africa, such as those in Lake Victoria that may provide a level of geographic isolation while having ecological diversity equivalent to continental sites. Such settings are shown to contain vector populations with low to moderate genetic differentiation and greater structure, suggesting limitations to migration [56]. However, not all African countries have access to such islands and the default exclusion from the conduct of initial trials solely on this basis would appear to be disproportionate and short-sighted. Alternatively, spatial, climatic, or seasonal isolation also could be used to reduce or retard the outward migration of gene drive mosquitoes from initial release locations [12]. Yet another approach, thus far restricted to modelling studies, could involve genetic isolation. This could be achieved by exploiting “locally fixed alleles” in island populations [57]. A “double drive” design could also be used, with interacting constructs inserted at two different locations in the genome, one of which is primarily responsible for the desired effect, population suppression or population modification, and the second responsible for the drive of the first [58, 59]. Modelling shows that such designs can restrict the spread and impact of the construct even if there is a relatively small level of differentiation between target and non-target populations.

As would be the case for low-threshold gene drive, field releases of *Wolbachia* and classical biological control agents also are intended to result in their indefinite spread and persistent in the environment. They therefore offer useful precedents for proportionate and appropriate considerations of the potential for reversibility of low-threshold gene drive when considering field trial locations and designs. Decisions to approve both the initial field trials and subsequent more widespread use of *Wolbachia* and numerous biological control agents have depended on neither geographic, nor other forms, of isolation, nor on the availability of mechanisms for reversibility, and instead have relied upon thorough ERA before release. For example, the first releases of *Wolbachia*-infected mosquitoes to control dengue took place only after rigorous ERA but without physical isolation on mainland Australia [60]. The neotropical parasitoid *Apoanagyrus lopezi* (Hymenoptera: Encyrtidae) was obtained from South America, then shipped to the United Kingdom for quarantine, before being released 125 times between 1981 and 1995 in 22 countries in continental Africa, without physical isolation, to successfully control the cassava mealybug, *Phenacoccus manihoti* (Hemiptera; Pseudococcidae) [61]. Other more recent examples of field releases in Africa of biological control agents that have not involved any physical isolation include: (i) the parasitoid wasp *Habrobracon hebetor* Say (Hymenoptera: Braconidae) being released in Burkina Faso to control the millet head miner, *Heliocheilus albipunctella* de Joannis (Lepidoptera: Noctuidae) [62]; (ii) the parasitoid wasp *Cotesia vestalis* (Hymenoptera: Braconidae) being released into Kenya to reduce populations of the diamondback moth *Plutella xylostella* (L.), (Lepidoptera: Plutellidae) [63]; (iii) the parasitoid *Acerophagous papayae* Noyes and Schauff (Hymenoptera: Encyrtidae) being released to control the papaya mealybug, *Paracoccus marginatus* Williams and Granara de Willink (Hemiptera: Pseudococcidae), which invaded Kenya in 2016 causing yield losses of 57–91% and annual household economic losses of circa $2800 per hectare [64].

*(5) Potential for reversibility*

There are several theoretical possibilities for the reversal of a released gene drive system, the feasibilities of which could in future be explored further experimentally in the laboratory. For example, in population suppression gene drive, where a genomic target disrupts fertility, there would be strong selective pressure for resistant alleles [10]. Mosquito strains could possibly be generated that contain polymorphisms at those genomic target loci making them refractory to homing, leading to their positive selection in the field, thus preventing the persistence and spread of the population suppression gene drive system in target populations. Strains with such polymorphisms could either be obtained by direct genetic editing techniques in the laboratory, or without any genomic editing or genetic modification via their selection in insectary cage studies. The use of “anti-drive” is also being explored as an approach to reduce rates of homing and gene drive in a population where a gene drive system has been released [65–67]. Another possibility could be to consider insecticide-based remediation approaches for the first field trials of low-threshold gene drive [12, 53].

*Holistic decision-making*

Ultimately, decision-making on specific locations, and designs, for the first field trials of low-threshold gene drive will necessitate the balancing of all five of the above broad criteria against each other as part of a process of iterative dialogue between developers, regulators, funders, communities living in field trial locations, and other stakeholders.

## Trial design

Key considerations for the trial design will include whether or not to include both release locations, where the gene drive system would be released, and control locations, where it would be absent, preferably over the entire course of the study [12, 68]. For example, in longitudinal trials of entomological efficacy where data collected at release locations before and after release of the gene drive system, confounding factors such as changes in rainfall or malaria transmission could augment or obscure before-and-after comparisons on the impact of the gene drive system on target mosquito populations [15]. In that regard, and depending on its objectives, a ‘controlled before-and-after’, or ‘pre-post control group’, trial design could be considered for such initial gene drive trials. Here, data are collected before and after the introduction of the intervention in both the experimental group receiving the intervention and control group that does not receive the intervention [15]. Such a design has been used to assess integrated malaria vector control using ITNs and microbial larvicides in Western Kenya [15, 69].

Cluster randomized controlled trials (cRCTs) [18], in which households, villages, or broader geographical areas (“clusters”) are randomly assigned either to the intervention or to the control, are considered to provide the most robust estimates of entomological and epidemiological efficacy with the least opportunity for the introduction of bias from confounding factors, although technical or ethical considerations in some vector control trials may necessitate the use of alterative designs [12, 15, 16, 18, 70, 71]. cRCTs have previously been used in numerous vector control field trials [15, 16], such as those involving ITNs [72] and other insecticide-based interventions [15, 16, 73–75] including eave tubes [76], or spatial repellants [77–80], ivermectin mass drug administration [81, 82], the *w*Mel strain of *Wolbachia pipientis* (Rickettsiales; Anaplasmataceae) [83–85], and attractive-toxin sugar bait traps [86–88].

The key elements to influence the power of a cRCT are (i) the number of clusters, (ii) the number of individuals within each cluster, (iii) the magnitude of the baseline measurement endpoint, (iv) the minimum difference in measurement endpoint between control and intervention arms for which the trial is powered, (v) the variation between clusters in the measurement endpoint, and (vi) the necessary separation distance between clusters to minimize “spillover effects” of the intervention between study arms [15, 18, 50]. Because primary outcomes in vector control trials are often based on epidemiological endpoints, they have typically been designed with statistical power to detect epidemiological, rather than entomological, impacts [69, 76, 86–89].

Where genetic efficacy would be the sole efficacy objective of initial field trials, this could potentially be assessed in observational studies without control locations, given the pre-release absence of the gene drive system from wild target mosquito populations, provided potentially confounding factors such as rainfall were also recorded [15]. Therefore, at the simplest level, initial field trials could involve release locations as “point sources” so that measurement endpoints would comprise the rate of increase in frequency of the gene drive system in target mosquito populations, as well as its linked traits affecting vectorial capacity, and the rate and variability of its spread to distal locations (Fig. 1).

Unlike current interventions, one of the attractions of the low threshold gene drive as a malaria vector control tool is that it is designed to spread and persist indefinitely in target populations. Hence, once these initial genetic efficacy trials were complete, it could be anticipated that the gene drive system would continue to spread and persist indefinitely in target mosquito populations. Given these properties, the released gene drive system could eventually reach control locations, effectively spilling over and “converting” those initial control locations to “passive” release locations in subsequent stages of the trial [18]. Consequently, where only genetic efficacy was to be assessed in initial field trials, locations of subsequent trials assessing entomological or epidemiological efficacy would need to be sufficiently distant to initial release locations to avoid the risk of spillover.

Where the objectives of an initial field trial were to assess entomological and epidemiological efficacy subsequent to genetic efficacy, at least two different strategies could be considered. In the first, the outcomes of the initial genetic efficacy field trial could inform the design of a later, and entirely separate, cRCTs measuring entomological and epidemiological endpoints, whereby the initial study locations would have to be sufficiently distantly located from the study locations of the subsequent trial to avoid the potential for spillover effects from the first trial to the second [18]. While the genetic efficacy outcomes from the first trial could differ to some degree from genetic efficacy outcomes in the subsequent trial, they would still provide an important scale of effect for later trial design.

In the second strategy for trials measuring genetic, and entomological, and epidemiological efficacy, the initial field trial could be designed as a single, integrated cRCT, which from the inception of its the genetic efficacy phase, would involve random allocation of clusters as release or control locations that would subsequently be used for entomological and epidemiological endpoints. In this case, a key constraint in a cRCT would be that all study locations would need to form part of a framework of random allocation [18]. To both accommodate the potential uncertainty in outcomes from the initial genetic efficacy phase of the trial and to allow optimization of the final design of subsequent entomological and epidemiological efficacy phases, such a trial could be adaptively designed [12, 68] to incorporate some flexibility in the duration of the genetic efficacy phase of the trial, albeit potentially increasing the risk of spillover. Therefore, in this kind of strategy, subsequent to genetic efficacy measurements both entomological and epidemiological endpoints would likely be assessed in parallel, with the entire field trial most likely being statistically powered for epidemiological primary endpoints [15].

**Fig. 4:**
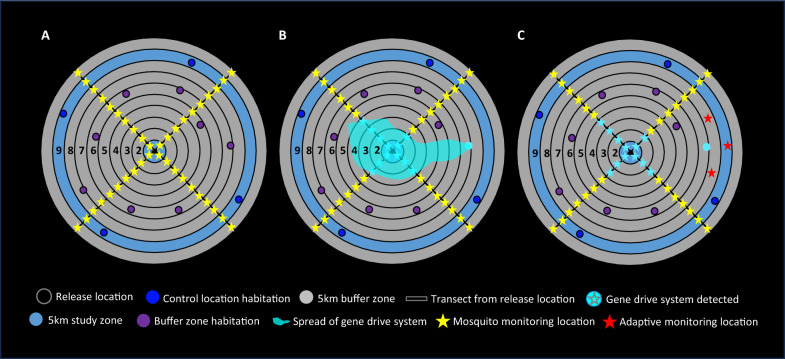
Illustrative scenario for monitoring of the spread of a gene drive system from a release location.** A** In this example, each ring (black circle) is a 5 km radial distance from the release location (black circle at centre). The area between rings is defined as an annulus, identified here by sequential numbering from the release location. The annulus surrounding the release location is the release zone (light-blue ring surrounding black circle of release location). For the purposes of this illustration, it is assumed that modelling indicates that the rate of spread of the gene drive system is 20 km per annum from the release zone. The duration of this initial field trial would be 2 years. In this particular trial design scenario, entomological and epidemiological measurements could be compared between the release location and control locations (four navy blue circles) in ninth annulus (medium-blue). A buffer zone of the second to eighth annuli (grey) minimizes the potential for ‘spillover’ of the gene drive system between the release and control locations. For the purposes of measuring spread of the gene drive system, either larvae or adult mosquitoes could be tested for the presence of the transgene at monitoring locations every 5 km along transects running northwest to southeast and northeast to southwest from the release location (yellow stars), as well as in buffer zone habitations (purple circles). **B** In an example of a potential adaptive trial design element for monitoring, the gene drive system is detected in mosquitoes at monitoring locations the first, second, third, and fourth annuli (light blue stars) but also at a buffer zone habitation in the seventh annulus (light blue circle) **C** Additional adaptive monitoring locations (red stars) are introduced at the eighth and ninth annuli between the two transects to ensure as accurate as possible monitoring of both the rate and variability of spread of the gene drive system

Transitions in the gene drive system status of control locations to release locations could potentially be accommodated by a type of cRCT design known as a stepped-wedge trial, in which an intervention is progressively rolled out across all trial locations or clusters [15, 18]. For example, a stepped-wedge trial design was used to assess the epidemiological impact on malaria of solar-powered odour-baited mosquito trapping systems in Kenya [90] and has been explored in the design of dengue vector control trials [91], although in the case of a low-threshold gene drive system, its spread from experimental to control locations would not formally be a randomized process. A so-called “fried egg” design has been employed in some cRCTs to allow primary outcomes to be measured within a core area from each cluster to limit spillover impacts from the intervention into controls [18, 88]. A related approach could employ sufficient distance, or “buffer zones” (Fig. 4), between release and control locations to limit the possibility that the gene drive system would spread to control locations over the course of the initial field trial, provided that such control locations retained similarity to release locations for entomological, environmental, ecological, and epidemiological attributes, which could be ensured through restricted randomization [18, 50]. Trials can also be designed to account for spillover between release and control locations via statistical design elements in addition to the use of buffer zones [14, 15, 18, 84, 85]. A recent modelling study has shown that results of seminal *Wolbachia* cRCTs demonstrating significant control of dengue transmission may have actually underestimated the true efficacy of the intervention because of three spillover effects caused by (1) human movement, (2) mosquito movement, and (3) disease transmission coupling between treatment and control clusters, indicating the importance of modelling in design and interpretation of future trials of low threshold gene drive for malaria vector control [92]. Additionally, statistical analyses of data collections from locations along *gradients* of changing frequency of the gene drive system, from release locations through buffer zones to control locations, could also be highly informative in testing the effectiveness of the causal pathway.

In trial design, “blinding” is the concealment of the allocation of clusters to experimental or control arms from investigators and study participants [18]. This is desirable whenever possible to minimize the introduction of bias into site selection, data acquisition or reporting, or from differences human behaviour at control and experimental locations. Where full blinding is not feasible, it may still be possible to conduct portions of the trial, for example laboratory analyses, under blinded conditions [18].

Box 3. Mosquito sampling and detection methods The choice of sampling methods and their effective application is important in facilitating accurate determination of both genetic and entomological efficacy of the gene drive system in target mosquito populations, as well as potential impacts on non-target populations. A variety of methods can be used to obtain mosquito samples for the measurement of species abundance, or frequency or spread of the gene drive system. Sampling methodologies include aspirators, pyrethrum spray catches (PSC), or U.S. Centers for Disease Control and Prevention (CDC) light traps, Biogents (BG) sentinel traps, swarm sampling for adult mosquitoes, or larval dipping for aquatic stages. Specific choices on the types and range of sampling methods may depend on the area, target population, and objectives of the initial field trials. For example, female mosquitoes can be captured indoors and outdoors using aspirators, PSC or CDC light traps. Stationed pit shelters, tent traps, swarm catches, and BG sentinel traps can be used to sample mosquitoes outdoors [102].

While the human landing catch (HLC) has been referred to as “the gold standard” for measuring biting rates of mosquitoes both indoors and outdoors, its use can be resource-intensive and, importantly, raises ethical challenges regarding the increased exposure of study volunteers to infectious bites [103]. This has prompted investigation of alternatives methods, such as mosquito electrocuting traps (METs), Furvela tent traps (FTTs), host decoy traps (HDTs), and outdoor CDC light traps (OLT) [103].

Where the location and distribution of aquatic habitats of target populations is well-understood, larval dipping could be an efficient and systematic way to detect gene drive system frequency in both males and females. There have been a number of recent developments in the use of Unmanned Aerial Vehicles (UAVs; “drones”) combined with machine learning approaches to identify mosquito aquatic habitats; these approaches could make larval dipping more informative as a sampling method for species density measurements and transgene detection in both target and non-target populations [104–108].

Any limitations of specific types of methodologies could lead to potential sampling biases, which together with natural variation and stochasticity in mosquito numbers, could impact the thresholds with which gene drive mosquitoes might be detected in the field. Inadequate levels and distribution of monitoring could also yield anomalous data where the spread of the gene drive system does not occur uniformly, or is accompanied by the re-population of wild-type mosquitoes, for example in case of the emergence of resistance to drive, or underestimates of the spread of the gene drive system from the failure to detect it at locations distal to the release site, pointing to the need for adaptive approaches to monitoring for the first field trials. All of these factors can guide the development and refinement of statistically based sampling strategies that can shape monitoring protocols that take into consideration sampling bias, natural variation, and detection thresholds.

Molecular assays on genomic DNA from field-caught samples are likely to be used to determine the frequency of transgenics in target populations and in those of sibling species (Figs. 3 and 4) [109–111]. The choice of detection method for the gene drive system in the field is also likely to affect operational flexibility and efficacy assessments (Fig. 3). For example, while gene amplification analyses, such as PCR, are highly sensitive to the detection of specific DNA sequences, including for species identification, they are also prone to contamination [112]. PCR also depends on availability of specialized laboratory infrastructure and technical expertise that is not ubiquitous at remote study sites. In order to make field operations more impervious to contamination, it is worth considering the use of alternative technologies such as colorimetric LAMP- or ELISA-based assays [113]. Where fluorescent reporter transgenes form part of gene drive system, it could be feasible to readily detect the gene drive system in field-caught mosquito samples by direct observation of expressed fluorescent reporter proteins via field-adapted florescence detection devices [114].

As well as monitoring gene drive system frequency, field-caught mosquito samples also could be subjected to molecular analyses to investigate whether any polymorphisms or mutations can be detected at the genomic target locations of gene drive systems, which may be indicative of the emergence of resistance to drive. Likewise, the potential for resistance to the anti-Plasmodium effector gene could be examined in samples of the parasite. One promising approach that is under active development, but which has yet to achieve appropriate robustness, sensitivity, and specificity, could be the use of environmental DNA (eDNA) to assess the frequency of the gene drive system, or perhaps even species abundance, particularly in aquatic habitats. For example, in case of population suppression gene drive, reductions in the densities of target populations might lead to reductions in the densities of predators or increases in in the densities of non-target competitor species [23]. Here, sampling and detection of eDNA from in aquatic habitats, in combination with quantitative PCR methods [115, 116], could be used to assess the abundance of such species of mosquito target populations, potential in conjunction with UAV technology [106, 107, 117].

Numerous AI, machine learning, and deep learning approaches are also currently being developed to allow the identification, and potentially counting [107], in the field of mosquito species at adult [117–127] and aquatic stages [127], using morphological characteristics, or even wingbeat patterns [126], which could be augmented by DNA barcoding analyses [128]. These approaches might also be combined with the use of UAVs to conduct mosquito vector population surveillance with minimal human resource implications [107]. Should these approaches deliver on their promise in a timely manner, they could be highly useful additions to more conventional sampling and detection tools that are already available for use in initial field trials for low threshold gene drive.

## Adaptive trial design

**Fig. 5:**
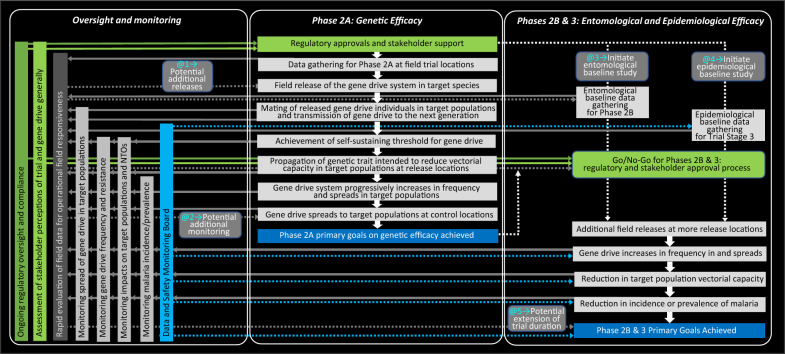
Illustrative example of a potential adaptive trial design for initial field trials of low threshold gene drive. Considering the intended potentially indefinite persistence and spread of the gene drive system in target populations, the first field trials could be divided into two Phases, building on the WHO guidance framework for the evaluation of GMMs [12]. Phase 2A would initially focus on the primary goals of assessing the increase in frequency of the gene drive system at, and the rate of its spread from, the release location but would also involve initiation of baseline data collections for subsequent assessment of Phase 2B entomological (‘@3’) and Phase 3 epidemiological (‘@4’) endpoints. Phase 2A of the trial could involve release of the gene drive system in a single vector species or multiple ones. Should the primary goals of Phase 2A be achieved, Phases 2B and 3 could be activated to allow the parallel assessment of entomological endpoints and epidemiological endpoints, potentially with expanded releases of the gene drive system in a single vector species or multiple ones Additional adaptive trial design elements could include flexibility in the numbers of mosquitoes and frequencies of release to achieve self-sustaining transmission of the gene drive system (‘@1’), adapting monitoring (‘@2’; see also Fig. 4); and potential to extend the duration of the trial where efficacy and safety assessments support this (‘@5’)

The attributes of indefinite spread and persistence of low-threshold gene drive prompt consideration of alternative types of more flexible trial design, or “adaptive trial design”, than those used for more conventional vector control interventions [93–97] (Fig. 5). In the context of clinical trials of pharmaceuticals, biologics, and health system interventions, adaptive trial design first gene drive field trial protocol incorporates the possibility of incremental escalation to more elaborate or complex goals once initial primary goals had been successfully achieved.

On one level, adaptive design for gene drive research programmes could involve initial phases that separately allowed assessment of modular components of a gene drive system that would precede the assessment of a fully integrated low threshold gene drive system [12]. For example, the components of a gene drive system could be tested without the need, or even the possibility, for their propagation beyond the immediate trial area by using modular systems where the gene drive and anti-pathogen effector gene functions are separate genetic components [98–101]. If earlier phases involving non gene drive components were successful, such a genetically confined approach would then proceed to later phases featuring self-sustaining gene drive (see Table 1).

On another level, developers could seek provisional and conditional regulatory approval and stakeholder support for a comprehensive protocol beginning with small-scale releases to test genetic efficacy in Phase 2A, then progressing to an expanded set of releases to assess entomological (Phase 2B) and epidemiological efficacy (Phase 3) subject to satisfactory genetic efficacy outcomes [12] (Fig. 5). Nevertheless, if additional locations were to be enlisted as the trial progresses, then their assignment as release or control clusters would need to be random; alternatively, groups of additional locations could be randomized to release or control clusters and treated as a stratum [18]. Importantly, if additional locations were assigned on the basis on interim analysis of data, the imposition of more stringent p-values on primary endpoints might be needed [18].

In such a scenario, formal permission for the first releases in Phase 2A would initiate an assessment of the increase in frequency of the gene drive system in target populations and its spread from the release locations, with the simultaneous activation of baseline entomological measurements and recruitment of human participants for a baseline assessment of epidemiological efficacy at Phases 2B and 3 of the trial (Fig. 5 ‘@3’ and ‘@4’). Successful outcomes from the Phase 2A of the trial would then activate Phases 2a and 3 of the trial involving additional field releases and parallel assessments of entomological and epidemiological endpoints. An alternative scenario could involve the sequential, rather than parallel, assessment of entomological and epidemiological efficacy in the trial, whereby successful entomological outcomes in Phase 2B would then activate the assessment of epidemiological endpoints in Phase 3.

In either of the above two scenarios for adaptive field release trials, the initial release locations could only form part of a cRCT if they are part of the random allocation of sites to either intervention or control. One way that this could be achieved would be to begin the field trial with a small, even number of locations and group these in pairs, so that the two members of each pair were as similar as possible to each other and then randomly allocate locations within each pair to release or control arms of the trial. For example, initially the gene drive field trial could commence with only one pair of locations, with the initial releases taking pace in the randomly selected release location. Additional pairs of study locations could be added as the size and complexity of the trial expanded, but always with random allocation of locations to release and control arms of the trial, until there was a sufficient build-up of clusters to a fully powered cRCT. In this way, entomological and epidemiological comparative evaluations of the two trial arms could commence once outcomes from earlier genetic efficacy evaluations were incorporated into their optimal design and implementation. By monitoring the spread of the gene drive system from the release to control locations from the beginning of the trial at the earliest randomly allocated pairs, the appropriate size of the buffer zone between locations within pairs could be determined, so that locations with pairs that would be subsequently added to the trial could be sufficiently separate from one another to avoid spillover.

## Assessing genetic efficacy in Phase 2A

Given the pre-release absence of the gene drive system from wild populations, an obvious ambition in initial field trials will be to investigate genetic efficacy endpoints. Observation of an increase in the frequency of the gene drive system in target populations after cessation of releases would provide one aspect of the successful demonstration of genetic efficacy. This could be based initially on the measurement of the frequency of the gene drive system in mosquitoes at the release locations and proximal environs over an extended period (Fig. 3 and Box 3). It would be useful to understand the basis for any differences between the predicted increase in frequency of the gene drive system and those observed empirically in the field.

Potential factors that could disrupt this include unanticipated fitness costs in gene drive mosquitoes, low rates of homing by the gene drive system in the field, or the rapid evolution of resistance to gene drive [129–131]. These potential disruptors should inform what is monitored and ensure that lower-than-expected efficacy can be investigated using data from those first trials to improve future designs of gene drive systems. Assessment of genetic efficacy would occur over multiple generations post-release, as well as accounting for seasonal variability, particularly changes in rainfall that alter larval habitat availability, or larval migration via surface water movement that might influence the spread of the gene drive system. For example, the gene drive system might impose fitness costs so that the gene drive mosquitoes were less likely to survive the dry season, or simply be stochastically lost during this period due to the low densities of target mosquito populations arising from poor availability of aquatic habitats [132, 133].

Previous field studies of GMMs [12] have typically involved the release of male mosquitoes to avoid the potential for increased rates of biting on human or animal hosts that would be caused by augmentation of the number of wild-type female mosquito vectors by released female GMMs [12, 114, 134]. Immediately following the release of male gene drive mosquitoes, they would need to mate with and transmit the gene drive system to wild field mosquitoes. Alternatively, if the number of individuals to be released was relatively low compared to wild-type mosquito populations, females that had already been mated in the insectary could be considered for releases because this would reduce the release ratio of gene drive mosquitoes required to yield the same increase in frequency of the gene drive system over time compared to releases of males. Regardless, the released mosquitoes may incur fitness costs that only manifest under field conditions that were not detected in laboratory fitness assays. Inhibition of transmission of the gene drive system could arise because of (i) disruptive insectary manipulation and transport of the gene drive strain to field sites, (ii) spatial or temporal misalignment of releases with target mosquito populations, (iii) unintended fitness costs due to the gene drive system itself, or (iv) effects of the genetic background of the strain of mosquitoes carrying the gene drive system; such a strain will have become adapted to environmental conditions within laboratory cages and therefore may be less fit than wild mosquitoes in the field, including any of its progeny for several generations post-release. Nonetheless, fitness costs associated with laboratory adaptation may be less of a hindrance to onward transmission of a gene drive system because its homing in in the germline of target populations at the release locations should result in its rapid increase in frequency. Careful rearing in the insectary and preparation of mosquitoes to be released should reduce the possibility of deleterious effects of transmission of the gene drive system from manipulation, transport, or release conditions. Crossing of gene drive mosquitoes to wild types that have recently been derived from the field populations could be used to mitigate genetic background fitness costs [135].

The impacts of potential fitness costs of the initially released gene drive mosquitoes also could be overcome by built-in flexibility in field trial protocol via adaptive trial design (Fig. 5) [93, 95], whereby releases were continued until the gene drive system was established in mosquito target populations at a frequency that was self-sustaining. For example, the homing rate of the gene drive system in the field, which might differ from that observed in the laboratory, could be estimated following the release of male gene drive mosquitoes by measuring the ratio of offspring with and without the gene drive system from gravid field-captured females. In this scenario, by collecting gravid females directly from field, the proportion of females that mated with gene drive males could be determined and compared with observed proportion of gene drive males obtained in swarm catches by mark-release-recapture (MRR) [134]. If this were to coincide with the initial release, the mating competitiveness of released males, evaluating fitness costs associated with a combination of laboratory adaptation, genetic background, and transgene impact, could be evaluated. If after initial releases, so that all males with the gene drive system in the field have arisen from post-release generations, fitness costs, including mating competitiveness, from rearing effects will no longer be a factor for consideration, nor will laboratory adaptation and genetic background fitness costs as they reduce with each generation, so that any reduced fitness would be limited to the gene drive system itself. Regardless, the most direct and relevant data will be the change in frequency of the gene drive system over time, which could be augmented by examining different life stages to infer differential effects from homing rates, specific fitness effects, and generation times.

Another measure of genetic efficacy would be assessment of the progressive spread over time in target populations of the gene drive system from release locations. This could be made by measuring the radial distance at which the gene drive system can be detected from its release locations (Fig. 4), including over an extended period post-release that would encompass multiple mosquito generations and any seasonal changes in rainfall that alter larval habitat availability and migration via surface water movement, thus affecting the potential for spread. Initial estimates of spread could be informed by close-kin MRR studies [136], with kinship estimates based on single nucleotide polymorphisms [137], spatial genetics [138], traditional MRR trials [134], and modelling [12, 55]. Decisions on the distribution and frequency of monitoring locations along transects could also be informed by these methods, as well as modelling [139], and potentially the further development of insecticide resistance molecular surveillance programmes [140, 141]. The spread of the gene drive system would likely deviate from concentricity from the release site for a variety of reasons, such as potential local variations in population structure or spatial variability in species composition leading to assortative mating, or geographic barriers, as well as from variable patterns of dispersal caused by windborne migration, human routes of travel, availability of water, blood and nectar sources, flooding that might carry aquatic stages over long distance, and stochasticity [142–144]. Considerations on the measurement of spread of gene drive systems could include selection of transects extending bidirectionally from release locations through buffer zones and to control locations where the gene drive system would be known to be absent [145] (Fig. 4). Final choices could be informed by estimates of spread of the gene drive system using data from earlier genetic studies, or MRR trials, using high recapture technique combined with modelling. Given that spread and persistence in target populations of low-threshold gene drive systems are its intended features, adaptive trial designs with responsive monitoring strategies may also be considered, whereby one could extend the intensity and spatial range of monitoring, if gene drive system were to be detected at the outer limit of the initial monitoring area that had been predicted from modelling (Figs. 4 and 5).

Genetic efficacy assessment would also require evaluation of the simultaneous propagation of a linked genetic trait aimed at reducing vectorial capacity for *Plasmodium* as it increases in frequency and spreads in target populations, an aspect that differs between population suppression and population modification approaches. For example, for a population suppression gene drive approach currently under investigation, the gene drive system disrupts the *doublesex* gene required for female fertility [146, 147]. *doublesex* is haplosufficient so that females that are heterozygous for the gene drive system are fertile, but sterile as homozygotes. Hence, the intended impact on vectorial capacity using this approach is to reduce the density of females in target mosquito populations. In addition, females that are homozygous for the *doublesex*-based gene drive system have anatomical alterations to their proboscis, rendering them unable to blood feed [146]. Hence, as this gene drive system increases in frequency in target mosquito populations, field-caught homozygous females would be readily identified morphologically, and their fertility status subsequently assessed. For population modification approaches, in addition to components required for homing, the gene drive system could likely also encode genes designed to reduce or prevent parasite transmission [148], so that the intended impact on vectorial capacity is to reduce the vector competence of target populations for *Plasmodium*. As the gene drive system increases in frequency and spreads in target populations, either the sporozoite rate, or the potential for parasite infection in field assays, could be compared in field-caught females with and without the gene drive system [36–38, 70, 74, 81, 149, 150]. Genetic efficacy assessment would also include monitoring for stability of the gene drive system including in the case of population modification for the potential of the functional effector gene to become uncoupled from the homing mechanism. The gene drive system also could be sequenced to directly assess whether the effector genes meant to reduce vector competence have been maintained.

Overall, it should be possible to assess the genetic efficacy of a gene drive system, namely its increase in frequency, spread, and simultaneous propagation of a linked genetic trait aimed at decreasing vectorial capacity for malaria parasites, in small scale field trials involving only one or two release locations and without the need for control locations, unless part of a larger cRCT as described earlier. This is because the unit of analysis here will be the individual mosquito, rather than the population.

## Assessing entomological efficacy in Phase 2B

The WHO recommends the gathering and assessment of baseline entomological data from study locations that are relevant to efficacy, such as vector species composition and rates of malaria transmission, which can incorporate variation in endpoints over multiple seasons and transmission cycles [12, 68]. It also proposes that questions identified in ERA should inform the types of baseline data that will be required to characterize the impact of the field releases on health and the environment. Furthermore, it points out that mathematical modelling and network analyses also could identify significant species interactions that will inform baseline data gathering. Considerations could include: (i) key ecological data, (ii) mosquito and parasite genetic variation, (iii) seasonal variation in vector species densities, (iv) fitness parameters such as fertility, fecundity, and survival, (v) locations of aquatic habitats, (vi) locations of swarming sites, (vii) levels of migration of the target wild type mosquitoes, and (viii) mosquito biting behavior [12]. In addition, potential confounding factors that could alter mosquito densities or malaria transmission at the trial locations also should be considered as part of baseline measurements, including (ix) human impacts on the landscape such as habitation, irrigation, and agriculture, (x) rainfall, temperature, climate, and geography, and (xi) existing or planned vector control measures (Fig. 3). These confounding factors can be addressed by appropriate randomization of locations to control and release arms of the field trial.

In contrast to genetic efficacy, entomological efficacy would be assessed using populations as the unit of analysis and would typically occur once a gene drive system has reached, or is close to reaching, fixation in target populations (Figs. 1 and 3; Box 3). To maximize the potential for the first gene drive field release trials to be as informative as possible, specific research questions and endpoints should be chosen for which anticipated effect sizes are as large, and variability as low, as possible (Table 1). Decisions on trial design also could be based on observational mechanistic linkages within the causal pathway, for example, correlation of increased frequency of the gene drive system with reduced entomological inoculation rate (EIR) in target vector populations. However, previous guidance from the Vector Control Advisory Group (VCAG) of the WHO recommends statistically powering entomological endpoints for trials [15].

For population suppression gene drive, comparison of the density of target populations of mosquitoes at release and control locations will be an important entomological parameter to assess [70, 71, 74, 75, 81, 90, 151]. For example, a battery of ovitraps and BG sentinel traps covering both control and release locations were used to ascertain measurements relating to adult mosquito density in a trial of GMMs to control *Aedes aegypti* in Brazil [114]. For population modification gene drive, comparison of sporozoite rate of females in target populations at release and control locations will be a key assessment, in addition to associated biting rates and densities in the same populations to provide an overall measurement of EIR [70, 71, 74, 81]. In theory, non-target vector species could serve as entomological controls in field studies; however, species composition can vary considerably across nearby locations and seasonally and the intervention itself could, for example, affect competitive interactions between vector species [23]. The fraction of total EIR that could be reduced as a result of gene drive in the target species could then be estimated from baseline data. Use of genetic information in mosquito target populations, such as measurement of nucleotide diversity or linkage disequilibrium, may also provide an additional and useful method of gauging the effectiveness of population suppression gene drive, as such genomic parameters depend on population size [152].

As there is likely to be significant variation in densities of target populations across study locations and over time due to variation in environmental factors such as rainfall as well as sampling biases imposed by collection methods [15, 109, 153–155], field implementation of a statistically powered assessment of entomological endpoints could also require considerable resources. Although entomological outcomes are not by default reliable predictors of epidemiological outcomes, such entomological measurements could contribute high-quality data that should lead to important correlations with any observed epidemiological impacts [17, 156, 157]. Accurate and representative entomological data collection from field sites that have been selected as potential release or control locations would be highly valuable in informing whether entomological outcomes from initial field trials are attributable to the gene drive system and distinct from stochastic background variation [12].

VCAG has suggested that six to ten randomly selected households or trap nights per site, with entomological measurements compared in a time series over the course of a season rather than as a simple before-and-after measurement [15]. Drawing on baseline field entomological data, statistical tools are under active development that can inform decision-making on choices of entomological endpoints, and to inform power calculations on releases in single versus multiple vector species for initial field trials of low-threshold gene drive. As with the design of any cRCT, the amount of variation in entomological measurements between trial locations also will be a factor in determining the number of clusters required in control and intervention arms of the field trials involving low threshold gene drive [15, 50, 158–160]***.***

In addition to entomological endpoints measuring efficacy, any entomologically based risks identified in pre-release ERA of the gene drive system will most likely also need to be assessed at this phase of initial cRCTs. These could include measurements evaluating whether (i) there were any adverse effects from detection of the gene drive system in other sibling species sympatric to the target mosquito populations, (ii) there were any effects on non-target organisms that adversely impacted health or ecosystem services, (iii) in the case of release of a population suppression gene drive, there were any effects on competitor or predator species of target mosquito populations that adversely impacted health or ecosystem services, (iv) in the case of population modification, there were any changes in pathogenicity or transmissibility of *Plasmodium falciparum* or in other species of malaria pathogens, such as *Plasmodium vivax*, *Plasmodium ovale*, or *Plasmodium malariae* that adversely impacted health or ecosystem services, or (v) there was any evidence of gene flow into non-reproductively compatible species of insect and that caused adverse impacts (Table 1).

## Assessing epidemiological efficacy in Phase 3

As with entomological efficacy, epidemiological efficacy would be assessed using the population or cluster as the unit of analysis once a gene drive system has reached, or is close to reaching, fixation in target populations. While successful implementation of the initial field trials of low-threshold gene drive systems may be favored when objectives are simple and focused on addressing essential efficacy and safety questions, such as whether the system can increase in frequency and spread in target populations, initial trials should also provide sufficient information, experience, and confidence to optimize field releases for later, more complex, and larger field trials. Similar incrementally expanding approaches are used as standard practice in Phase I-IV clinical trials of new pharmaceuticals or biotechnology products [13, 161].

Definitive epidemiological assessments or results could be confounded by other vector control interventions being used in the study area. VCAG has recommended that trials should be statistically powered for epidemiological endpoints [15]. Epidemiological efficacy of the gene drive system may be more difficult to establish, but easier to implement, when relying solely on passive case detection of malaria prevalence [12, 15, 162], as this measures the rate of both pre-existing and new symptomatic infections in the population, and is prone to variability in the health-seeking behavior of people, the quality of diagnostic services available, and administrative practices, such as record-keeping and attributions of correct residential location to patients. Therefore, while it remains possible that information on cases of malaria at release and control locations of the first gene drive field trials could be captured by passive case detection at health centers or in routine surveillance programmes, the prevalence of infection would be more robustly assessed by means of a representative randomly selected cross-sectional survey, usually a household survey, in all clusters, by measuring infection status of individuals [12, 70, 75, 76, 162] (Fig. 3).

Such data would be relevant for both population suppression and population modification approaches. In addition, active case detection is more likely to have the power to detect significant changes in the incidence of infection and disease [12, 81, 90, 163]. However, this approach might be considered as a more expensive and time-consuming investment in initial field trials as it would most likely require the recruitment and treatment of participants with anti-malarial medications to eliminate any pre-existing cases of parasitaemia ahead of the commencement of the gene drive releases and assessment. Nonetheless, such recruitment is routinely the case in other cRCTs for malaria vector control. Therefore, given that the fundamental objective in the development of low-threshold gene drive in *Anopheles* mosquitoes is to reduce malaria transmission, active case detection of malaria incidence would likely produce robust and accurate assessments of epidemiological efficacy [12].

Additional aspects of morbidity associated with malaria, such as anaemia and splenomegaly, could also be assessed in trial participants at control and release locations [70, 75] (Fig. 3), although in most published cRCTs these endpoints typically seem insufficiently sensitive to changes in malaria transmission. The relationship between parasite prevalence and clinical incidence of malaria appears to be more complex than a simple linear one, so that simultaneous measurements of both these endpoints might be contemplated in ideally designed vector control trials [50, 164].

In addition to epidemiological endpoints measuring efficacy, any epidemiologically based risks identified in pre-release ERA of the gene drive system will most likely need to be assessed in this stage of initial cRCTs***.*** These could include measurements evaluating whether there are changes in (i) the incidence or prevalence of malaria before and after field releases or at release versus control locations, (ii) the incidence or prevalence of other diseases before and after field releases or at release versus control locations, (iii) human behavioural responses towards conventional vector control measures such as bednets as a result of releases of the gene drive system, or (iv) perspectives of the local or wider communities to the specific intervention and wider gene drive technology (Table 1).

While the time to impact of releases of gene drive systems for malaria vector has been investigated in a number of modelling studies, most of these have focused on post-trial implementation that have typically involved annual releases of low numbers of mosquitoes at relatively dispersed locations yielding time to entomological or epidemiological impacts on the order of one to two years after releases have commenced [23, 99, 165]. However, a recent modelling study of initial field releases of a population modification gene drive system on an island setting suggested that, under at least some circumstances, a significant reduction in malaria incidence could be anticipated within three to six months [148]. The development of further modelling tools and studies exploring a range of different release rates, frequencies, and spatial structures should inform expectations on how rapidly efficacy might be observed in initial gene drive field trials. Another important consideration may be whether one particular kind of gene drive may be more effective than another in specific malaria transmission settings. This in turn may affect the outcomes to be measured. Indeed, the epidemiological impact may depend on the vector species being targeted by the gene drive system and the species composition in the study area (Box 2)***.***

It also could be possible in adaptive field trial design to extend the duration of the trial depending on emerging data (Fig. 5 ‘@5’). For example, in the case of population modification gene drive, the gene drive system could continue to spread from the release location without its effector gene [166]. For population suppression gene drive, mosquito populations may be reduced but not eliminated, allowing the gene drive to continue to spread through target populations [165].

Such examples underscore the importance of the outputs of ERA of the release of the gene drive system in playing a key role to allow regulatory and stakeholder decision-making around the acceptability of the potential risks versus the potential benefits from a low-threshold gene drive system, including when and if the trial should be considered to have ended. Nonetheless, any flexibilities in the duration of the trial would have to be subject to clearly defined stopping rules, which could impose penalties of lower p-values for statistical significance any time an interim analysis were to be carried out [18]. In addition, some form and level of post-trial field monitoring of the gene drive system, at the release locations and potentially distally, may be required by decision-makers [12].

Considering the indefinite persistence and spread of low-threshold gene drive in the field, whether initial field trial focus exclusively on measurement of genetic efficacy, or on broader aspects of the causal pathway, how and when epidemiological efficacy will eventually be assessed will be an essential consideration before decisions on any field trial protocols are finalized and implemented.

## Conclusions

A fundamental question for field trials of low-threshold gene drive for malaria vector control will be whether the intended causal pathway leading from release of gene drive mosquitoes to reduction in malaria transmission will be effective. Key decisions for initial trials will focus on whether primary objectives should focus exclusively on assessments of genetic, entomological, or epidemiological efficacy, or combinations thereof. While effective conduct of initial field trials of low-threshold gene drive systems may be favoured when objectives are simple, such as whether the system can increase in frequency and spread in target populations, initial trials also should be designed and implemented to provide sufficient breadth and depth of information, experience, and confidence to optimize field releases for later, more complex, and larger field trials involving more robust epidemiological assessments. Because spread and persistence of low-threshold gene drive systems in target populations are intended features, adaptive trial design offers a potentially constructive and flexible approach to incremental testing of the causal pathway. Such an approach could facilitate the first field trials of low threshold gene drive to be staged incrementally so that genetic efficacy would first be assessed and, if proven, activate further stages of testing involving entomological or epidemiological endpoints, or both. How and when epidemiological efficacy will eventually be assessed will be an essential consideration before decisions on any field trial protocols are finalized and implemented, regardless of whether initial field trials focus exclusively on measurement of genetic efficacy, or on broader aspects of the causal pathway. Statistical and modelling tools that will support decision-making on choices of such trial locations and endpoints, adaptive trial design, robust power calculations, effective monitoring tools and methodology, and field operational responsiveness to emerging monitoring data during trials, are currently under active development. The considerations here advance the realization of developer ambitions for the first field trials of low-threshold gene drive for malaria vector control within the next 5 years. 

## Data Availability

Not applicable.

## Abbreviations

ACT: Artemisinin-based combination therapy
AUDA-NEPAD: African Union Development Agency-New Partnership for Africa's Development
BG: Biogents™ (sentinel traps)
CDC: U.S. Centers for Disease Control and Prevention
cRCT: Cluster randomized controlled trial
DSBM: Data and Safety Monitoring Board
eDNA: Environmental DNA
EIR: Entomological inoculation rate
ELISA: Enzyme-linked immunosorbent assay
ERA: Environmental risk assessment
FNIH: Foundation for the National Institutes of Health
GCP: Good Clinical Practice
GMM: Genetically modified mosquito
HLC: Human landing catch
ICH: International Council for Harmonization
IRS: Indoor residual insecticide spraying
ITN: Insecticide-treated bed net
LAMP: Loop-mediated isothermal amplification
LLIN: Long-lasting insecticidal bed net
MRR: Mark-release-recapture field study
PCR: Polymerase chain reaction
PSC: Pyrethrum spray catch
UAV: Unmanned Aerial Vehicle (“drone”)
VCAG: Vector Control Advisory Group of the WHO
WAIVM: West African Integrated Vector Management Programme
WAHO: West African Health Organization
WHO: World Health Organization
WMA: World Medical Association
