# Publisher Correction: Considerations for first field trials of low-threshold gene drive for malaria vector control

**DOI:** 10.1186/s12936-024-05049-z

**Published:** 2024-08-05

**Authors:** John B. Connolly, Austin Burt, George Christophides, Abdoulaye Diabate, Tibebu Habtewold, Penelope A. Hancock, Anthony A. James, Jonathan K. Kayondo, Dickson Wilson Lwetoijera, Alphaxard Manjurano, Andrew R. McKemey, Michael R. Santos, Nikolai Windbichler, Filippo Randazzo

**Affiliations:** 1 Department of Life Sciences, Silwood Park, Imperial College London, London, UK; 2 Department of Life Sciences, South Kensington Campus, Imperial College London, London, UK; 3 Institut de Recherche en Sciences de la Santé/Centre Muraz, Bobo-Dioulasso, Burkina Faso; 4 Environmental Health and Ecological Science Department, Ifakara Health Institute, Ifakara, Tanzania; 5 MRC Centre for Global Infectious Disease Analysis, St. Mary’s Campus, Imperial College London, London, UK; 6 Departments of Microbiology & Molecular Genetics and Molecular Biology & Biochemistry, University of California, Irvine, USA; 7 Entomology Department, Uganda Virus Research Institute (UVRI), Entebbe, Uganda; 8 Malaria Research Unit and Laboratory Sciences, Mwanza Medical Research Centre, National Institute for Medical Research, Mwanza, Tanzania; 9 Foundation for the National Institutes of Health, North Bethesda, MD USA; 10 Leverage Science, LLC, Berkeley, CA USA

**Correction: Malaria Journal (2024) 23:156** 10.1186/s12936-024-04952-9

Following publication of the original article [1], it came to the journal's attention that there were errors in references to Figs. 2 and 3. Some references to Fig. 2 should have been a reference to Fig. 3, and vice versa. The article has since been updated to correct these errors. The publisher thanks you for reading this erratum and apologizes for any inconvenience caused.
